# Dynamic Cerebral Autoregulation Changes during Sub-Maximal Handgrip Maneuver

**DOI:** 10.1371/journal.pone.0070821

**Published:** 2013-08-14

**Authors:** Ricardo C. Nogueira, Edson Bor-Seng-Shu, Marcelo R. Santos, Carlos E. Negrão, Manoel J. Teixeira, Ronney B. Panerai

**Affiliations:** 1 Department of Neurology, Hospital das Clinicas, University of São Paulo School of Medicine, São Paulo, Brazil; 2 Department of Neurosurgery, Hospital das Clinicas, University of São Paulo School of Medicine, São Paulo, Brazil; 3 Heart Institute (InCor), University of São Paulo Medical School, São Paulo; School of Physical Education and Sport, University of São Paulo, São Paulo, Brazil; 4 Medical Physics Group, Department of Cardiovascular Sciences, University of Leicester, Leicester Royal Infirmary, Leicester, England; 5 Biomedical Research Unit in Cardiovascular Science, Glenfield Hospital, Leicester, England; University of Arizona, United States of America

## Abstract

**Purpose:**

We investigated the effect of handgrip (HG) maneuver on time-varying estimates of dynamic cerebral autoregulation (CA) using the autoregressive moving average technique.

**Methods:**

Twelve healthy subjects were recruited to perform HG maneuver during 3 minutes with 30% of maximum contraction force. Cerebral blood flow velocity, end-tidal CO_2_ pressure (PETCO_2_), and noninvasive arterial blood pressure (ABP) were continuously recorded during baseline, HG and recovery. Critical closing pressure (CrCP), resistance area-product (RAP), and time-varying autoregulation index (ARI) were obtained.

**Results:**

PETCO_2_ did not show significant changes during HG maneuver. Whilst ABP increased continuously during the maneuver, to 27% above its baseline value, CBFV raised to a plateau approximately 15% above baseline. This was sustained by a parallel increase in RAP, suggestive of myogenic vasoconstriction, and a reduction in CrCP that could be associated with metabolic vasodilation. The time-varying ARI index dropped at the beginning and end of the maneuver (p<0.005), which could be related to corresponding alert reactions or to different time constants of the myogenic, metabolic and/or neurogenic mechanisms.

**Conclusion:**

Changes in dynamic CA during HG suggest a complex interplay of regulatory mechanisms during static exercise that should be considered when assessing the determinants of cerebral blood flow and metabolism.

## Introduction

The neurovascular response to exercise and augmented cerebral metabolic demand (neurovascular coupling) relies on dynamic adjustments of multivariate systems, involving myogenic, metabolic and neurogenic mechanisms that lead to constriction or dilation of cerebral arteriolar smooth muscles in order to control cerebral blood flow [1], [2]. This response is mediated by the neurovascular unit through activation of neuronal cells such as astrocytes and release of neurotransmitters [3]. This theory has led to the concept that during exercise there are continuous oscillations of the vascular tone to match cerebral blood flow to physiological needs [4], [5]. The handgrip maneuver (HG) is a static exercise consisting of contraction of forearm muscles. In healthy subjects HG leads to increases in heart rate (HR), arterial blood pressure (ABP) and cardiac output [6]. Whereas these changes are believed to be due to reflexes arising from stimulated muscles [7], other mechanisms, such as metabolic changes [8] and brain control, have also been proposed [9]. Recently it has been demonstrated that HG exercise also induces changes in cerebral blood flow (CBF), possibly due to bilateral activation of cortical brain areas implicated in muscle contraction and autonomic regulation [7], [10]. These effects, and concomitant changes in ABP, have allowed the HG maneuver to be used for assessment of dynamic cerebral autoregulation (CA) [10]–[15]. This approach assumes that HG maneuver in itself would not disturb CA, which seems to be supported by several studies [10]–[12], [14], [15]. However, one major limitation of most previous studies on this subject was the assumption that CA could be described by constant parameters, despite the physiological nonstationarity of the maneuver. To a large extent, this limitation resulted from techniques adopted to assess dynamic CA, such as transfer function analysis [10] or sudden release of compressed thigh cuffs [14].

To address the problem of nonstationarity of the HG maneuver, we implemented a new approach to obtain time-varying estimates of dynamic CA to test the hypothesis that CA does not remain constant throughout this maneuver. Autoregressive moving-average (ARMA) models have been used previously to obtain time-varying estimates of dynamic CA indices, which can then be correlated with peripheral and cerebrovascular beat-to-beat parameters to provide a more complete depiction of the complex interactions taking place during static exercise such as the HG maneuver [15], [16].

## Methods

### Subjects and measurements

The research ethics committee of the University of São Paulo (Brazil) approved this study; informed written consent was obtained from each subject and from the next of kin on behalf of the minors participants involved. Twelve healthy subjects (5 men) aged 39.4±19.5 (range 16–87) years old were recruited. Exclusion criteria comprised any history of cardiovascular and neurological diseases (including migraine), and lack of acoustic temporal bone window. Subjects were told to avoid alcohol, nicotine and caffeine-containing products 12 hours prior to attending the laboratory. Cerebral blood flow velocity (CBFV), ABP, and end-tidal carbon dioxide partial pressure (PETCO_2_) measurements were performed in a quiet and temperature controlled (22–23°C) room to minimize cognitive stimulation. Subjects were in the supine position with the heads slightly elevated by a pillow. ABP was recorded noninvasively from the left upper limb by an arterial volume-clamping device (Finometer™, Finapress Medical Systems BV, Netherlands) with the arm of measurement kept at heart level. PETCO_2_ was measured with an infrared capnograph (Dixtal, DX 1265 ETCO2 CAPNOGARD, Manaus, Brazil) via a closely fitting mask. Blood flow velocities in the right and left middle cerebral arteries were concomitantly measured using a transcranial Doppler device (DWL, Doppler-box, Germany) equipped with 2-MHz transducers which were placed over the temporal bone windows and held in place with a specially designed head frame. The insonation depths varied from 50 to 55 mm. Both ABP and CBFV data were transferred continuously to a computer for offline analysis. PETCO_2_ was calculated at 1 min intervals.

The HG maneuver was performed with a dynamometer. For each subject, maximum contraction force was calculated as the average of three rounds of maximum effort values with at least ten seconds of recovery between each task. In the main experiment, subjects were instructed to perform HG maneuver with the right arm at 30% of maximum contraction force for 3 minutes, and not to move any muscle other than those involved in the test [17]. After resting for at least 10 min, a single continuous 11 min recording was obtained corresponding to 5 min of baseline, 3 min of HG followed by 3 min of recovery.

### Data analysis

ABP and CBFV signals were sampled at a rate of 200 samples/s. All signals were visually inspected to identify artifacts or noise; narrow spikes (<100 ms) were removed by linear interpolation. All signals were low pass filtered with a cutoff frequency of 20 Hz. The beginning and end of each cardiac cycle were detected and mean beat-to-beat values were calculated for the right and left CBFV and the ABP channels. Critical closing pressure (CrCP) and resistance-area product (RAP) were obtained by the first harmonic method [18]. Spline interpolation, followed by resampling at 5 Hz were performed to obtain time-series with a uniform time base. The dynamic relationship between mean ABP and CBFV was calculated by an ARMA structure described previously [16]. Briefly, estimates of the autoregulation index (ARI), originally proposed by Tiecks *et al* [19], were obtained as a time-varying parameter by reducing the sampling rate interval to 0.6 seconds and shifting a 60 s moving window along the ABP and CBFV signals [16]. For each 60 s window, the ARMA model coefficients were used to generate the CBFV response to a step change in ABP. Using the 10 template curves proposed by Tiecks *et al* [19], the corresponding ARI value was extracted by least squares every 0.6 s interval. The ARI ranges from 0 (absence of autoregulation) to 9 (best observed CA). For each 60 s window, the estimated value of ARI was placed in the middle of the window thus implying that no estimates are available for the first and last 30 s of data. Changes in CBFV, ABP and HR were expressed in % of mean baseline values calculated during the 30 s preceding HG.

### Statistical analysis

**Figure 1:**
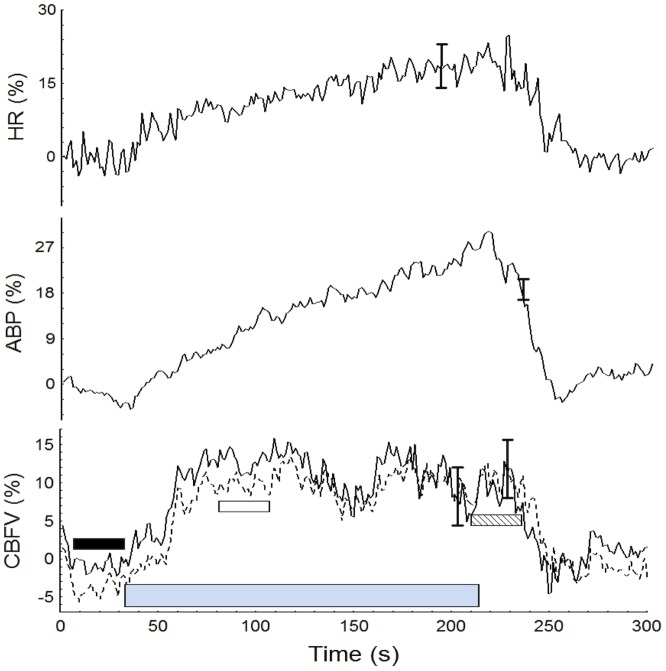
Population mean values of HR, ABP, CBFV_L_ (solid line) and CBFV_R_ (dashed line) synchronized by the beginning of HG. The grey bar represents the duration of HG. The small boxes at the bottom represent the time periods used to extract mean values at baseline (black box), P1 (blank box) and P2 (dashed box). For clarity only the largest ±1 SE is represented at the point of occurrence.

Repeated-measures ANOVA was used to test changes in PETCO_2_ at the baseline, at 1 min intervals during HG and at recovery. All beat-to-beat variables were synchronized at the beginning of HG and mean (coherent average) and standard deviation (SD) population values were calculated for each time sample at 0.6 s intervals. Coherent averages were also calculated during baseline using as point of synchronism the beginning of recording. Paired Student's t-tests were used to test right and left differences amongst all the variables and also to assess changes due to HG by comparing mean values during 10 s at the beginning of the response (P1, Fig. 1) against baseline values (B, Fig. 1). Repeated-measures ANOVA was adopted to assess the influence of late effects of HG on beat-to-beat variables by also considering the mean over 10 s at the end of the maneuver (P2, Fig. 1). Post-hoc analysis was performed with Schefeè's test. Points B, P1 and P2 were also used as reference to extract mean values of ARI for each subject, as well as the mid-maneuver value, that is 90 s after the start. Statistical significance was set at P<0.05.

## Results

**Figure 2:**
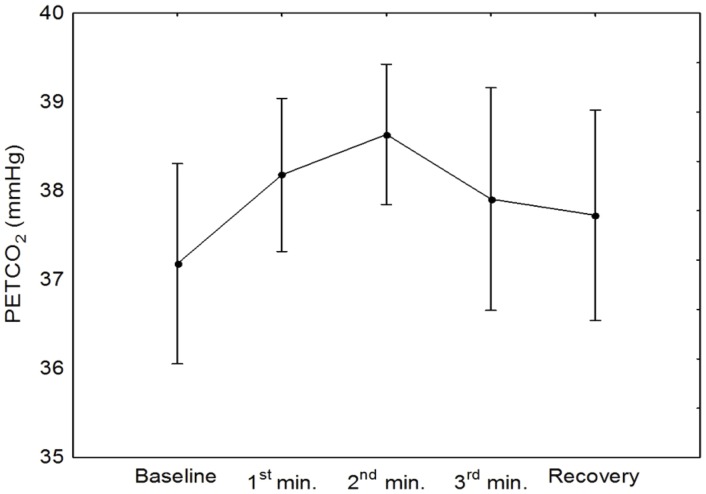
Mean (SE) of PETCO_2_ variation during the handgrip maneuver (ANOVA p = 0.330).

**Figure 3:**
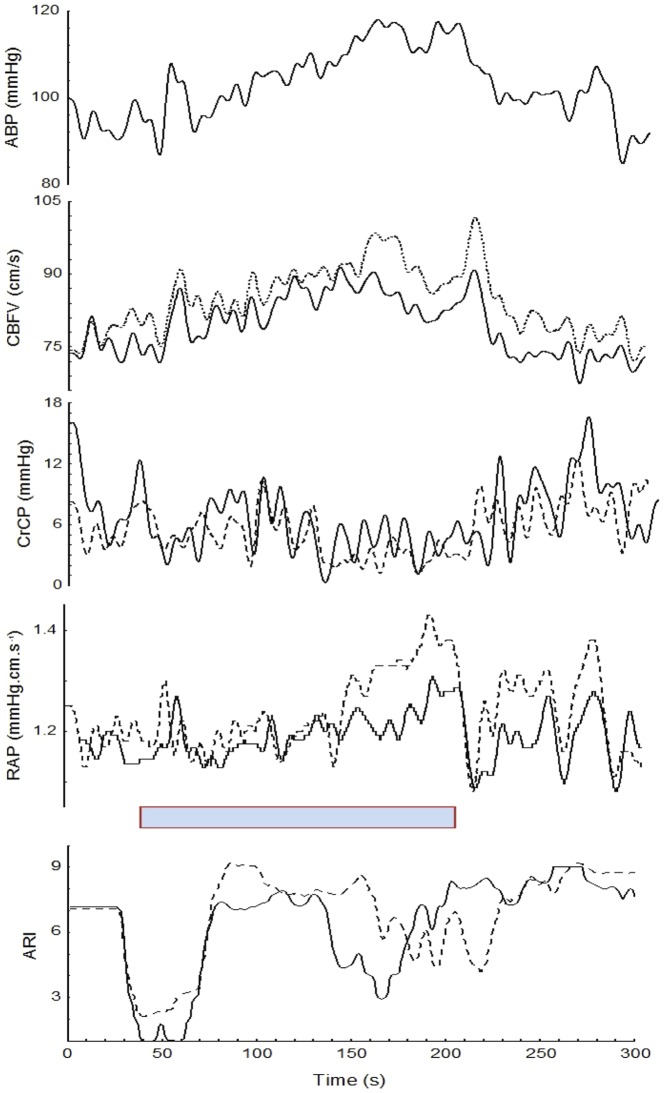
Representative pattern of the extracted and derived parameters in a 19 year-old female subject. The grey bar shows the duration of HG. From top to bottom: ABP, CBFV_L_ (solid line) and CBFV_R_ (dashed line), CrCP_L_ (solid line) and CrCP_R_ (dashed line), RAP_L_ (solid line) and RAP_R_ (dashed line), ARI_L_ (solid line) and ARI_R_ (dashed line). Subscripts R and L indicate right and left MCA respectively.

**Figure 4:**
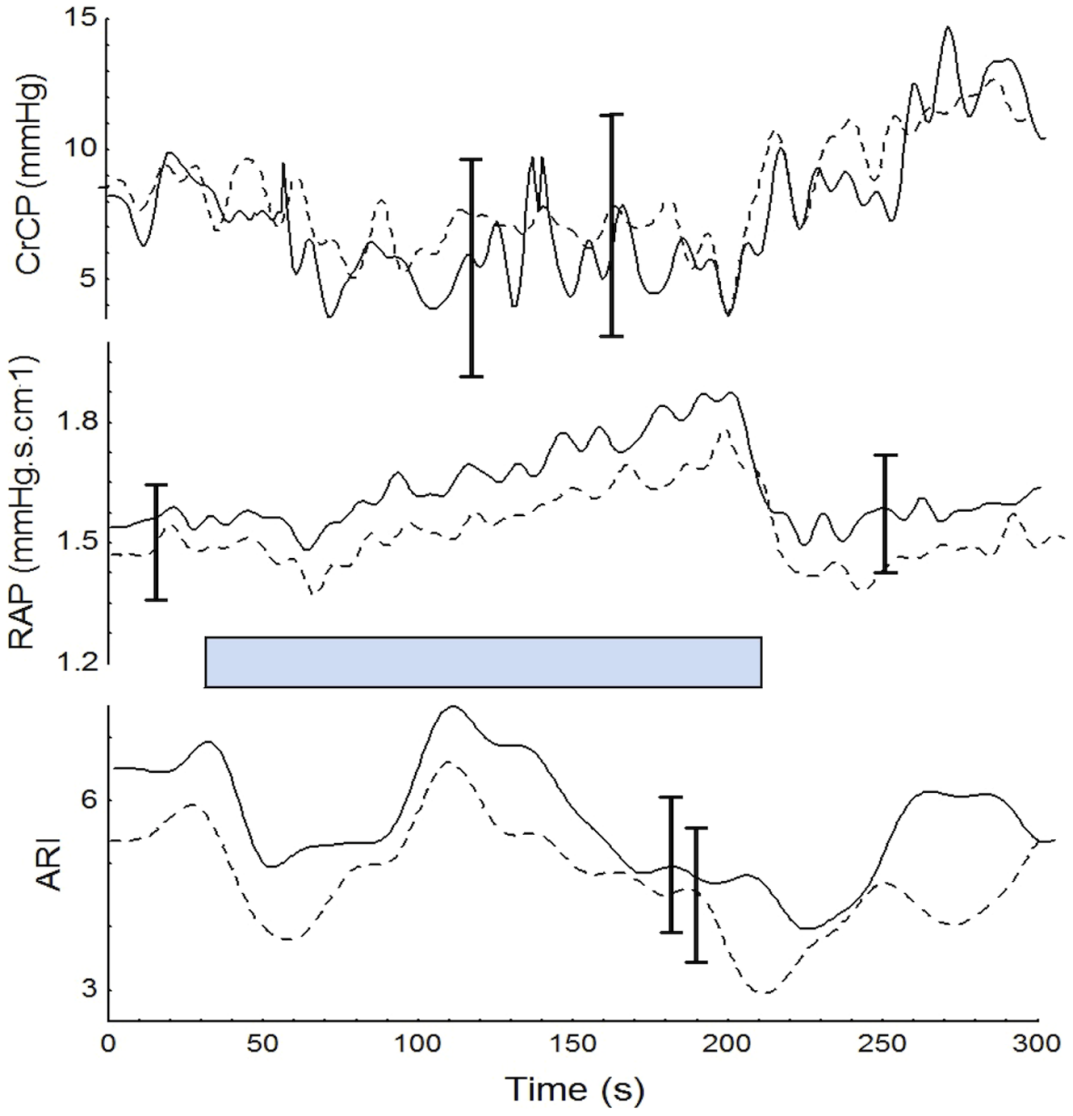
Population mean values of CrCP_L_ (solid line) and CrCP_R_ (dashed line), RAP_L_ (solid line) and RAP_R_ (dashed line), autoregulation index (ARI_L_: solid line and ARI_R_: dashed line). Subscripts R and L indicate right and left MCA respectively. For clarity only the largest ±1 SE is represented at the point of occurrence.

Table 1 provides the mean (SD) values of the recorded and derived parameters for the baseline and for the points P1 and P2 in the plateau phase. PETCO_2_ showed a trend to increase from baseline to the 2^nd^ min of the maneuver, however these changes were not significant (Fig. 2). Figs. 1 & 3 depict changes in ABP, HR, and CBFV during HG maneuver in good agreement to what was reported by other investigators. Corresponding changes from baseline to the plateau phases are given in Table 1. CrCP dropped significantly with the beginning of the maneuver (P1 vs B, Table 1), but the drop was not sustained as shown by the ANOVA (Table 1). On the other hand, changes in RAP associated with HG maneuver were characterized by a continuous rise reaching a peak before the end of the maneuver (Figs. 3 & 4 and Table 1).

**Table 1:** Parameter values of Baseline and Plateau during Handgrip maneuver.

	Baseline	Plateau 1	Plateau 2	P Value (ANOVA)
**CBFV_L_ (cm/s)**	60.4 (10.7)	68.8 (11.7)#	67.7(14.32)	0.003
**CBFV_R_(cm/s)**	65.0 (11.7)	74.0(13.7)#	73.0 (14.6)	0.000
**ABP (mmHg)**	98.6 (9.1)	112.9 (9.7)#	125.3 (9.5)	0.000
**HR (bpm)**	70.2 (10.9)	77.8 (9.8)#	83.2 (11.5)	0.000
**CrCP_L_(mmHg)**	8.09 (6.56)	4.69 (4.79)#	5.65 (5.37)	0.175
**CrCP_R_(mmHg)**	7.19 (6.91)	4.66 (5.26)*	6.01 (7.42)	0.593
**RAP_L_(mmHg.cm^−1^.s^−1^)**	1.55 (0.36)	1.63 (0.41)	1.83 (0.36)	0.013
**RAP_R_ (mmHg.cm^−1^.s^−1^)**	1.46 (0.34)	1.50 (0.27)	1.69 (0.33)	0.000
**CVR_L_**	1.68 (0.34)	1.69 (0.38)	1.93 (0.44)	0.003

Values are means (SD). CBFV, cerebral blood flow velocity; ABP, arterial blood pressure; HR, heart rate; CrCP, critical closing pressure; RAP, resistance area product; CVR, cerebrovascular resistance. Subscripts R and L indicate right and left respectively. P-values from repeated measures ANOVA.

# p<0.005,

* p<0.05 compared to baseline.

**Figure 5:**
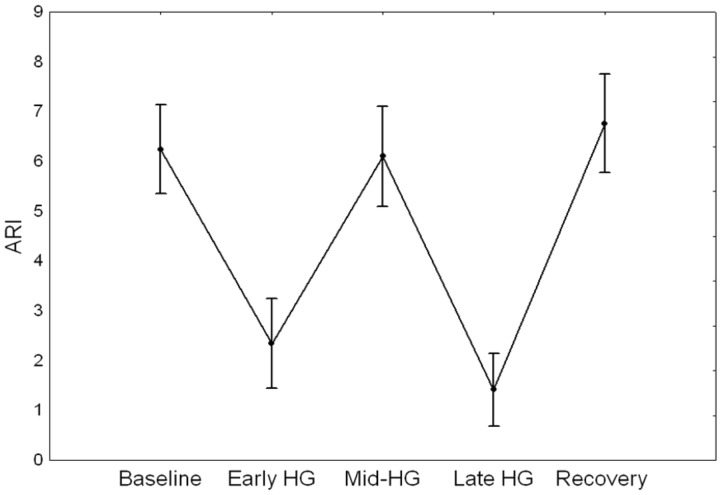
Mean (SE) of time-varying ARI at five distinct phases of HG.

**Figure 6:**
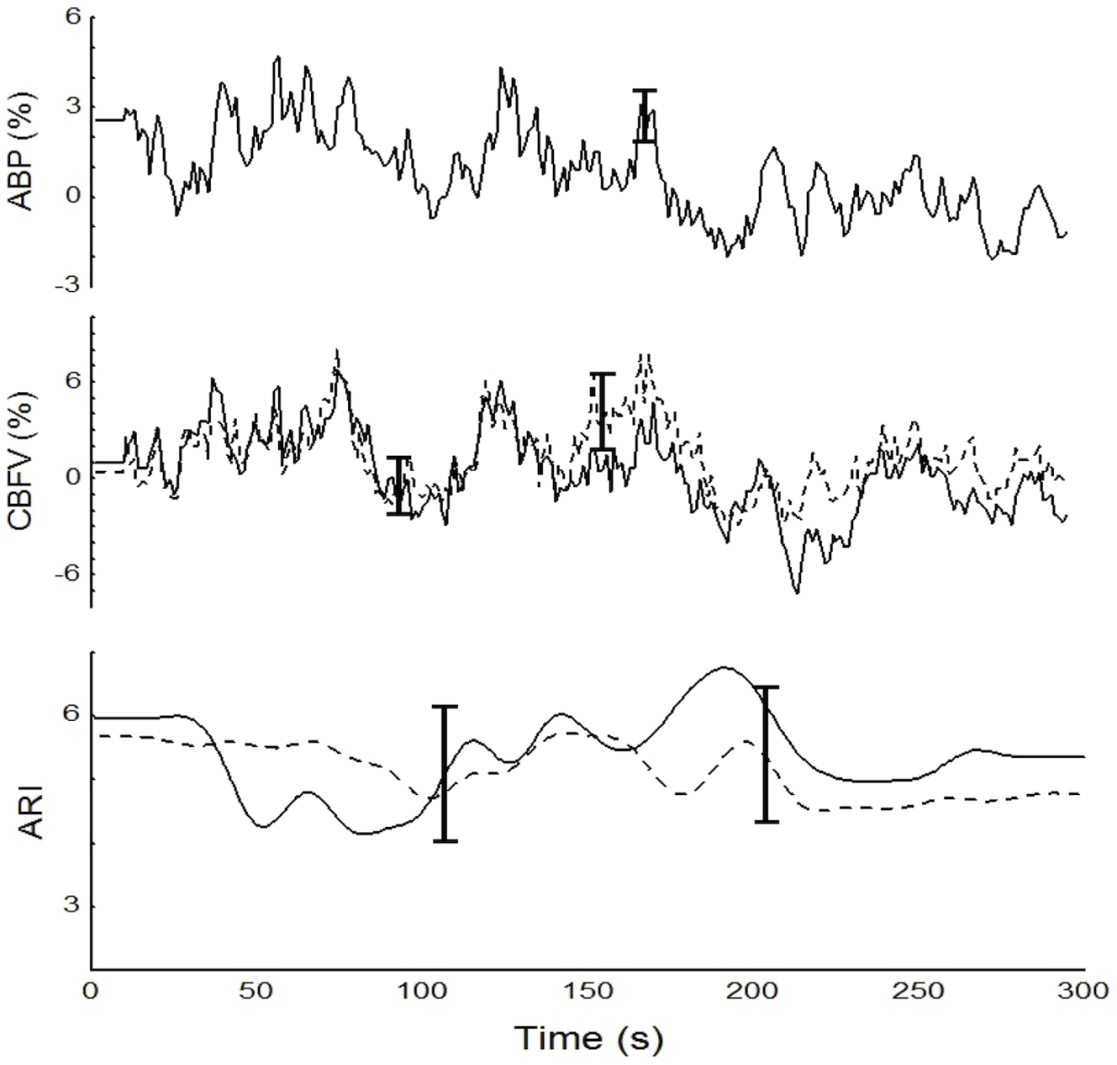
Population mean values during baseline synchronized by the beginning of recording. From top to bottom: ABP, CBFV_L_ (solid line) and CBFV_R_ (dashed line), ARI_L_ (solid line) and ARI_R_ (dashed line). Subscripts R and L indicate right and left MCA respectively. For clarity only the largest ±1SE is represented at the point of occurrence.

Time-varying estimates of ARI revealed highly significant changes during HG maneuver (ANOVA, p<0.001) (Figs. 3, 4 & 5). Post-hoc tests of ARI showed that the dips at the beginning and end of HG maneuver were significantly different from the baseline, mid-point and recovery phases (p<0.005 in all cases), but not different from one another. Temporal changes in ARI were also obtained during baseline (Fig. 6) albeit of smaller amplitude than observed during HG maneuver. For both HG maneuver and baseline conditions, visual inspection of individual recordings showed that 9 out of 12 subjects presented similar dips in ARI coinciding with those indicated by coherent averages (Figs. 4 & 6). There were no significant R-L differences for any of the variables studied.

The repeated measures ANOVA was also performed only in subjects <50 years old (n = 9) without any changes in inferences described above.

## Discussion

To the best of our knowledge, this is the first study concerning the application of ARMA modeling to generate time-varying estimates of dynamic CA during static handgrip exercise. Our main finding was that ARI, a widely used index of dynamic CA, was not constant during the HG maneuver; there were significant dips at the beginning and end of the maneuver. Surprisingly, similar, although less pronounced, dips were also observed during baseline.

A second important contribution of the study refers to the description of changes in CrCP and RAP during HG, which have not been reported to date. These variables provide a more realistic model of the instantaneous relationship between ABP and CBFV than the single variable model represented by cerebrovascular resistance [18], [20]. Previous work suggested that CrCP could represent microvascular adjustments, influenced predominantly by metabolic mechanisms, whilst RAP could reflect mainly myogenic activity [18], [21]. These hypotheses are partially supported by the present set of results. In response to HG maneuver, RAP followed the continuous rise in ABP, while simultaneous decrease in CrCP could be noted, the latter counteracting the effect of the former, and thus contributing to the relatively stable plateau of CBFV (Figs. 3 & 4). These results suggest the presence of conflicting mechanisms that interact to reach a new balance involving both vasoconstriction (RAP) and vasodilation (CrCP) of microcirculation during HG maneuver [4]. In other words, the HG maneuver led to a shift of the instantaneous relationship between ABP and CBFV [18]. Interestingly, a similar finding has been reported during pre-syncope [22], [23]. In addition, in-depth analysis of a recent study regarding lower limb exercises revealed that CrCP increases during heavy exercise, but tends to decrease during low intensity exercise (40% of maximum workload). Our own results on changes in CrCP (Fig. 4) are in agreement with these findings given that HG qualifies as low intensity exercise. This different behavior reinforces the contention that these variables are in constant change and should be assessed by nonstationary methods [24].

Concerning methodological issues, it has been difficult to assess the potential contribution of neurogenic mechanisms in the cerebrovascular response to the HG maneuver. Central nervous system has been implicated in the modulation of both systemic and cerebral hemodynamic responses to HG exercise, the latter possibly mediated by astrocytes, which are considered an essential component of the neurovascular unit [25]. Moreover, neural inputs have been suggested to influence the cerebrovascular response to HG maneuver [26]. These mechanisms may play an important role in the time-varying changes in dynamic CA, and deserve further investigation.

With ongoing changes in ABP, HR, breathing pattern, and possibly blood CO_2_ content, it would be surprising if dynamic CA remained constant during the HG maneuver. In fact, our results showed consistent ARI changes associated with the temporal course of the HG maneuver, not only in the mean population values (Fig. 4) but also on each individual recording (Fig. 3). The limitations of time-varying estimates of ARI using the moving-window ARMA technique have been addressed in previous reports and can explain the occurrence of sudden drops in ARI [27]. On the other hand, the physiological significance of the changes in ARI has been validated during respiratory maneuvers with the induction of hypo- and hypercapnia [16]. Despite the lack of overall significant changes in PETCO_2_ (Fig. 2), it has been demonstrated that small breath-to-breath changes in arterial P_CO2_ can induce fluctuations in ARI [28]. However, from previous results we hypothesize that the drop in ARI is more likely to result from the alert reaction produced by the beginning and end of the maneuver [16]. Other studies have also described impairment of dynamic CA due to stress [29]. New research protocols that could modulate the alert reaction are needed to test this hypothesis. An alternative explanation could be some degree of instability between the different mechanisms regulating CBF (myogenic, metabolic, neurogenic) compounded by different time constants, when these mechanisms are responding to multiple stressors as observed by the changes in ABP and HR at the beginning and end of the maneuver [4].

In contrast to our results, Ogoh *et al* [24], using the thigh cuff method for assessing cerebrovascular response to sudden drops in ABP, showed that the estimates of dynamic CA were similar during resting, HG exercise, and recovery conditions. However, during HG maneuver, the vascular conductance index curve during 10 seconds after cuff deflation showed a double pattern differently from those measured during baseline and recovery conditions, reinforcing the possibility that some instability of CA may have occurred during the maneuver. A possible explanation for these conflicting results could be the short length of time considered by Ogoh *et al* [24] for analysis (a few seconds during the maneuver), whilst in our study, drops in ARI were identified by assessing this parameter from beginning to end of the maneuver.

We performed coherent averaging of ARI during baseline with the expectation of finding much smaller fluctuations in ARI than those observed at the beginning of HG. We were surprised to find out that following the start of recording, the ARI also dipped at the same time that ABP and CBFV showed a transient rise (Fig. 6). The observation that CBFV is following ABP approximately in phase also suggests a time-localized reduction in CA efficiency. The fact that similar time coherent oscillations were observed in the majority of studied subjects (9/12) supports their physiological origin rather than random fluctuations. Again, our initial interpretation of this finding can be related to alert reaction at the beginning of recordings. Further studies of time-varying dynamic CA during resting conditions should explore potential co-factors, such as small spontaneous fluctuations in arterial P_CO2_ [28] due to alterations in breathing pattern.

This study has some limitations; the use of CBFV as a surrogate for cerebral blood flow can produce misleading results if there is variation of the cross-sectional area of the monitored cerebral artery [30]. There is evidence that the middle cerebral artery (MCA) diameter remains constant during increases in ABP and also arterial P_CO2_ [31] but there is less evidence with exercise or the HG maneuver. One particular study using a spectral index to estimate MCA flow during rhythmic HG claimed that the MCA diameter could be reduced by as much as 10% [32]. Another concern is the assumption that noninvasive ABP measurements in the finger are representative of the MCA perfusion pressure. A previous study showed that despite small differences, estimates of cerebral hemodynamic parameters and time-varying ARI from finger plethysmography measurements produce similar results when compared to those estimated using intra-arterial measurements in the ascending aorta [27]. However, it is possible that changes in peripheral vasomotor regulation can take place during HG in the contralateral limb, leading to distortions in the estimation of the ARI. Further investigation is needed, ideally using intra-arterial BP measurements in the ascending aorta during HG. The ARMA model can estimate the variation of ARI in time, but the first 30 s of each recording will be lost because of the 60 s duration of the moving window. This limitation is unlikely to have influenced our results because our subjects were monitored for at least 5 minutes during baseline. Finally, our population age range was intentionally wide to study both young and old subjects. In a recent study it was reported that the CrCP evaluated during dynamic exercise varies between young and old subjects [33], contrary to our study in which CrCP did not change significantly with ageing; it is possible that the type of exercise have influenced the results since our study employed static HG maneuver while Ogoh *et al* [33] employed dynamic exercise.

In conclusion, the study of dynamic CA in HG maneuver using the ARMA technique is feasible and could enhance our knowledge about changes in cerebral hemodynamics caused by static exercise. Longitudinal changes in CA parameters induced by HG exercise or other maneuvers can open new avenues of investigation into the regulation of CBF and also advance current clinical methods for assessment of patients with cerebrovascular disease.
